# A new genus *Squamophis* of Asteroschematidae (Echinodermata, Ophiuroidea, Euryalida) from Australia

**DOI:** 10.3897/zookeys.129.1202

**Published:** 2011-09-16

**Authors:** Masanori Okanishi, Timothy D. O’Hara, Toshihiko Fujita

**Affiliations:** 1 Department of Biological Science, Graduate School of Science, The University of Tokyo, Hongo 7-3-1, Bunkyo-ku, Tokyo, 113-0033 Japan; 2 Department of Zoology, National Museum of Nature and Science, 4-1-1, Amakubo, Tsukuba, 305-0005 Japan; 3 Museum Victoria, GPO Box 666, Melbourne 3001 Australia

**Keywords:** taxonomy, *Squamophis*, *Squamophis albozosteres*, new genus, new species

## Abstract

*Squamophis*, a new genus of brittle star is described. Two species are included in the genus: *Squamophis amamiensis* (Okanishi & Fujita, 2009) from south-western Japan and *Squamophis albozosteres* **sp. n.** from north-western Australia. *Squamophis* **gen. n.** is distinguished from the other genera of the family Asteroschematidae by the following characters: each radial shield is single-layered and is completely covered by plate-shaped epidermal ossicles, and the relative length of the longest arm spine throughout the arms is as long as the length of the corresponding arm segment. *Squamophis albozosteres* **sp. n.** is distinguished from *Squamophis amamiensis* in having white, slightly domed, plate-shaped epidermal ossicles on the aboral side of the body, the ossicles on aboral and lateral portion of the arms form transverse rows, and the other part of aboral side of disc and basal to middle portion of arms are brown but tip of the arms are light purple.

## Introduction

The family Asteroschematidae was erected by Verrill (1899) and currently comprises four genera, *Asteroschema* Örsted & Lütken, 1856 (in Lütken 1856), *Astrobrachion* Döderlein, 1927, *Astrocharis* Koehler, 1904, and *Ophiocreas* Lyman, 1869 (Fell 1960; Baker 1980). Recent morphological studies of internal ossicles indicated that some species of the genus *Asteroschema* appear more similar to *Astrocharis*, rather than the other species of *Asteroschema* (Okanishi and Fujita 2009, 2011a). However, the taxonomy of these species was left unresolved.

In this study, a new genus of Asteroschematidae is established for two species, including one that is new. A tabular taxonomic key to the five recent genera of Asteroschematidae is provided.

## Materials and methods

Three specimens of the new species were collected on Commonwealth Scientific and Industrial Research Organisation (CSIRO) survey SS05/2007 by R/V Southern Surveyor and are deposited in the Museum Victoria (MV). They were fixed onboard in 70% ethanol.

Ossicles from a paratype of the new species were isolated by immersion in domestic bleach (approximately 5% sodium hypochlorite solution), washed in deionised water, dried in air, and mounted on SEM stubs using double-sided conductive tape. The preparations were sputter-coated with gold-palladium and examined with a Jeol JSM-6380LV SEM.

**Figure 1.**
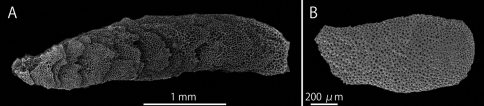
SEM photographs of radial shields of *Asteroschema tubiferum* (NSMT E-2110) (**A**) and *Squamophis albozosteres* sp. n., paratype(MV F-162658) (**B**). **A** multi-layered radial shield **B** single-layered radial shields. The left side of the images are towards the center of the disc and the right side towards the disc margin.

The terms used to describe asteroschematids follow (Okanishi and Fujita (2009, 2011a) and the terms used for the structure of ossicles follow Byrne (1994) and Martynov (2010). Some technical terms for internal dermal ossicles are newly defined in this study. Radial shields of most species of Asteroschematidae are composed of several thin, flat and plate-shaped ossicles (Fig. 1A), which vary in size, smaller toward the center of the disc and larger toward the periphery, and overlap slightly displacing each other (Fig. 1A). In this study, these radial shields are referred to as “multi-layered radial shields”. Each flat ossicle of the multi-layered radial shield is probably connected by soft connective tissue, thus when a multi-layered radial shield is dissected and immersed in domestic bleach, it disassembles into several ossicles. Other radial shields are composed of a single thin, flat and plate-shaped ossicle and are referred to as “single-layered radial shields” in this study (Fig. 1B).

The terms used for the superficial asteroschematid body ossicles have also been inconsistent in previous descriptions. Traditionally, “granules”, “scales” and “tubercles” have been used for the various ossicles on asteroschematid bodies (e.g.Mortensen 1933; Fell 1960; Baker 1980; McKnight 2000). In contrast, a recent histological study used the term “dermal granules” for ossicles on *Asteroschema oligactes* (Byrne, 1994). Use of “dermal ossicles” may be confusing because it would indicate both superficial and internal ossicles of echinoderms, including radial shields and vertebrae. Terms like “granules”, “scales” and “tubercles” give the impression that these ossicles are different kinds of ossicles. However, although asteroschematid ossicles can vary in shape, they are essentially the same kind of ossicle. In recent descriptions, we have used the terms “granule-shaped dermal ossicles”, “plate-shaped dermal ossicles” and “cone-shaped dermal ossicles” (Okanishi and Fujita 2009, 2011a, 2011b). Here, we suggest that these various shaped dermal ossicles should all be referred to as “epidermal ossicles”. “Epidermis” is the tissue that covers these superficial ossicles (Byrne 1994) and therefore the term “epidermal ossicle” indicates their superficial position and their difference to internal dermal ossicles. The relative size of ossicles is presented in terms of the length of the longest axis, and was measured using an ocular micrometer on a binocular stereoscopic microscope.

## Taxonomy

### Family Asteroschematidae

Verrill, 1899

http://species-id.net/wiki/Asteroschematidae

#### Type genus:

 *Asteroschema* Örsted & Lütken, 1856 (in Lütken 1856)

#### Type species:

 *Asterias oligactes* Pallas, 1788

#### Remarks.

Four genera are currently recognized within the Asteroschematidae: 1) the type genus *Asteroschema* erected for the Caribbean species *Asterias oligactes* Pallas, 1788 (=*Asteroschema oligactes*); 2) *Ophiocreas*, also erected for the Caribbean species, *Ophiocreas lumbricus* Lyman, 1869; 3) the genus *Astrocharis*, erected for Philippines’ species, *Astrocharis virgo* Koehler, 1904; and 4) *Astrobrachion*, erected for the New Zealand species, *Ophiocreas constrictus* Farquhar, 1900 (=*Astrobrachion constrictum*). The monotypic genus *Ophiuropsis* was erected by Studer 1884 for the Southwest African species, *Ophiuropsis lymani* Studer, 1884. This genus eventually contained one species with two subspecies, *Ophiuropsis lymani lymani* Studer, 1884 and *Ophiuropsis lymani simplex* Mortensen, 1933, but the former subspecies was junior synonymised with *Asteronyx loveni* Müller & Troschel, 1842 (Asteronychidae) and latter subspecies with *Astrobrachion constrictum* by Baker (1980). Mortensen (1933) erected a subgenus *Astrobrachion* (*Astroscolex*) for one of the two species of *Astrobrachion*, *Ophiocreas adhaerens* Studer, 1884 (=*Astrobrachion adhaerens*)but this taxon was also synonymised with *Astrobrachion* by Baker (1980).

In his key to the genera of Asteroschematidae, Fell (1960) used the following seven characters: 1) relative length of the arms to the disc diameter; 2) absence/presence of oral shields; 3) absence/presence of ventral arm plates; 4) variable covering of the radial shields; 5) shapes and arrangement of epidermal ossicles on the disc and arms; 6) relative length of the longest arm spine in throughout the arms to the corresponding arm segment; 7) and absence/presence of an abrupt increase in arm width between second and third, or third and fourth arm segments. Baker (1980) showed that two of these seven characters, the relative length of the arms to the disc diameter and absence/presence of oral shields were not useful. McKnight (2000) used another character, the degree of separation of the jaws, to distinguish the three Australian genera of Asteroschematidae, *Asteroschema*, *Astrobrachion* and *Ophiocreas*.

Our review of the taxonomic literature and examination of asteroschematid specimens, has indicated to us that several of these characters are not useful for defining genera. We have found that degree of separation of jaws varies in response to animal preservation. The abrupt increase in arm width, supposedly characteristic of *Astrocharis* (see Fell 1960) varies across asteroschematid species. An abrupt increase in width in basal portion of the arms can be observed in the original figures of the type species, *Astrocharis virgo* Koehler, 1904 and of *Astrocharis ijimai* Matsumoto, 1911. However, photographs in the holotype description of *Astrocharis gracilis* Mortensen, 1918 (in Mortensen and Stephensen 1918), which was synonymised with *Astrocharis ijimai* by Okanishi and Fujita (2011a), show no abrupt increase in width (Mortensen and Stephensen 1918; Döderlein 1927; Okanishi and Fujita 2011a) and *Astrocharis monospinosa* Okanishi and Fujita, 2011a also has no abrupt increase in width (Okanishi and Fujita 2011a).

We propose that four characters are useful for distinguishing the existing genera. The genus *Astrobrachion* has ventral arm plates separating the lateral arm plates on the oral midline throughout the arms, while the other genera have no ventral arm plates at least from the middle to distal portion of the arms. The genus *Astrocharis* has completely naked radial shields, whereas the radial shields of the other genera are completely covered by thick skin or epidermal ossicles. Therefore, the absence/presence of the ventral arm plates and the covering of the radial shields are useful generic diagnostic characters as Fell (1960) showed (Table 1).

**Table 1.** Tabular morphological key to the genera of the family Asteroschematidae.

Genus	Shape and arrangement of epidermal ossicles on aboral periphery of the disc and aboral basal portion of the arms	Radial shields	Ventral arm plate on middle to distal portion of the arms	Relative length of the longest arm spines to the corresponding arm segment
*Asteroschema* Örsted & Lütken, 1856*	Cone-shaped and completely in contact, or granule-shaped and slightly in contact	Multi-layered, covered by epidermal ossicles	Absent	Two times longer
*Ophiocreas* Lyman, 1869	Granule-shaped, slightly in contact or separated, or no epidermal ossicles	Multi-layered, covered by epidermal ossicles or skin	Absent	Two times longer
*Astrobrachion* Döderlein, 1927	No epidermal ossicles	Multi-layered, covered by skin	Present	The same length
*Astrocharis* Koehler, 1904	Plate-shaped and completely in contact	Single-layered, naked	Absent	Two times longer
*Squamophis* gen. n.	Plate-shaped and completely in contact	Single-layered, covered by epidermal ossicles	Absent	The same length

* Except *Astrocharis capense* and *Astrocharis igloo* which may be related to *Squamophis* gen. n. (see Okanishi and Fujita 2009, 2011a).

The shapes and arrangement of epidermal ossicles on aboral surfaces of the discs and arms have been used to distinguish the four genera (McKnight 2000), however, these ossicles vary in shape with location on the body so it is important only to compare ossicles from similar locations. For this study, we compared epidermal ossicles found on the aboral periphery of the disc and aboral basal portion of the arms, which recently appeared to be useful for distinguishing the species of *Asteroschema* and are expected to be useful for generic taxonomy (Okanishi and Fujita 2009). Although *Asteroschema* and *Ophiocreas* cannot be distinguished by this emended character (leaving aside the two species of *Ophiocreas*, *Ophiocreas gilolensis* Döderlein, 1927 and *Ophiocreas spinulosus*, Lyman, 1883, which have additional tubercle-shaped ossicles on the radial shields), the other two genera, *Astrobrachion* and *Astrocharis* can be clearly distinguished as follows: species of *Astrocharis* have only plate-shaped epidermal ossicles, and species of *Astrobrachion* have no epidermal ossicles (Table 1).

*Astrocharis* has been distinguished by its short arm spines (Fell 1960), but the longest arm spine is twice as long as the corresponding arm segment in *Astrocharis monospinosa* (Okanishi and Fujita 2011a). Since the relative arm spine length on *Asteroschema* and *Ophiocreas* species is approximately the same as that of *Astrocharis monospinosa*, these three genera cannot be distinguished from each other by this character (e.g. Döderlein 1911, 1927, 1930; Baker 1980; McKnight 2000). However, although not mentioned by Fell (1960), the length of arm spines on *Astrobrachion* species is indeed shorter than that of the other genera, being only the same length as the corresponding arm segment. Therefore, the four existing genera can be distinguished by this character as follows: the relative length of the longest arm spines throughout the arms is as long as the length of the corresponding arm segment in *Astrobrachion* but two times longer in the other three genera (Table 1).

### Genus Squamophis gen. n.

urn:lsid:zoobank.org:act:7470C786-4911-4D0B-A2E7-2D58742A3E2F

http://species-id.net/wiki/Squamophis

#### Type species:

 *Asteroschema amamiense* Okanishi & Fujita, 2009

#### Other included species:

 *Squamophis albozosteres* sp. n.

#### Diagnosis.

 Aboral periphery of the disc and aboral base of the arms covered completely by contiguous plate-shaped epidermal ossicles. Single-layered radial shields completely covered by epidermal ossicles. No ventral arm plates on middle to distal sections of the arms. Relative length of the longest arm spines the same length as the corresponding arm segment throughout the arms.

#### Etymology.

 The generic name is a masculine noun in the subjective case, a compound of Latin, *squama* (prefix, meaning “scale”) referring to the plate-shaped epidermal ossicles on their body and the Greek *ophis* (masculine noun, meaning “snake”), referring to their snake-like arms.

#### Remarks.

 (Okanishi and Fujita (2009, 2011a) examined internal ossicles of many species of *Asteroschema* and revealed that *Asteroschema amamiense* differed in having both single-layered radial shields and contiguous plate-shaped epidermal ossicles, on the aboral periphery of the disc and the aboral base of the arms, that are similar in shape and arrangement to species of *Astrocharis*. Furthermore, a recent molecular phylogenetic analysis (Okanishi et al. in press), based on mitochondrial (16S) and nuclear ribosomal RNA genes (18S, 28S), also showed that *Squamophis albozosteres* sp. n. (as *Asteroschema* sp.) and *Astrocharis monospinosa* form a clade that was separated from the two other species of *Asteroschema* that were sequenced, *Asteroschema ajax* A. H. Clark, 1949 and *Asteroschema ferox* Koehler, 1904. This new species also had single-layered radial shields and contiguous plate-shaped epidermal ossicles. However, both *Asteroschema amamiense* and *Squamophis albozosteres* differed from *Astrocharis* species in having covered radial shields and relatively short arm spines that are only as long as the corresponding arm segment. This morphological and molecular phylogenetic evidence suggests to us that these two species should be distinguished at a generic level from the other species of *Asteroschema* and *Astrocharis*. Therefore, we describe a new genus *Squamophis* for these two species. The distinguishing characters for the new genus are given in Table 1.

The genus *Squamophis* currently comprises two species: *Squamophis amamiensis* (Okanishi & Fujita, 2009) from south-western Japan, 167–168 m and *Squamophis albozosteres* sp. n. from north-western Australia, 95–108 m. *Asteroschema capense* Mortensen, 1925 and *Asteroschema igloo* Baker, 1980 may also be related to *Squamophis amamiensis*, based on the similarity of shapes and arrangement of epidermal ossicles (Okanishi and Fujita 2009). However, we have not examined the nature of radial shields on their type specimens and hence we refrain from transferring these two species to the new genus at this time.

### Squamophis albozosteres sp. n.

urn:lsid:zoobank.org:act:9D85F117-BE04-4BF6-A03A-68636966B737

http://species-id.net/wiki/Squamophis_albozosteres

Figs 1B 3 [Fig F4] [Fig F5] [Fig F6] 7 

#### Type materials.

**Figure 2.**
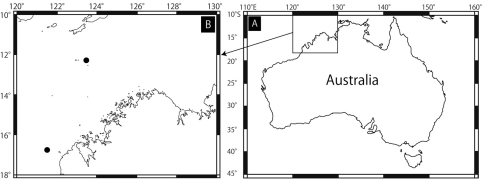
Collected sites of *Squamophis albozosteres* sp. n. Northern solid circle is for Ashmore Reef and southern one is off Broome.

MV F 162657, holotype, stn SS05/2007 116, off Broome, northwestern Australia, 16°45.09'S, 121°02.48'E – 16°44.36'S, 121°02.12'E, 100–108 m, rocky bottom, 23.3 °C, 30 Jun 2007, epibenthic sled. MV F162658, two paratypes, stn SS05/2007 188, off Ashmore Reef, northwestern Australia, 12°26.42'S, 123°36.03'E – 12°26.58'S, 123°36.35'E, 95–96 m, rocky bottom, 24,8 °C, 7 Jul 2007, benthic dredge (Fig. 2).

#### Diagnosis.

Epidermal ossicles conspicuous white, slightly domed and round plate-shaped, irregularly placed on aboral side of disc and forming two transverse bands on aboral and lateral sides of each arm segment. Rest of the aboral surface uniformly brown except light purple near the tips of arms and finally without color at the tip.

#### Description of holotype.

**Figure 3.**
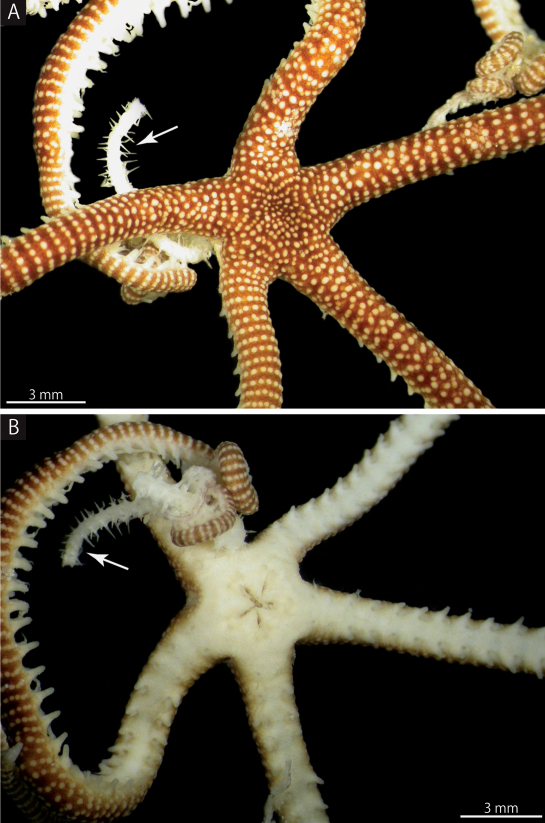
*Squamophis albozosteres* sp. n., holotype (MV F162657). **A** aboral view **B** oral view. Arrows indicate the arm of another ophiuroid gripped by *Squamophis albozosteres*.

MV F162657: disc diameter 3.4 mm, arm length approximately 50 mm (Fig. 3).

**Figure 4. F4:**
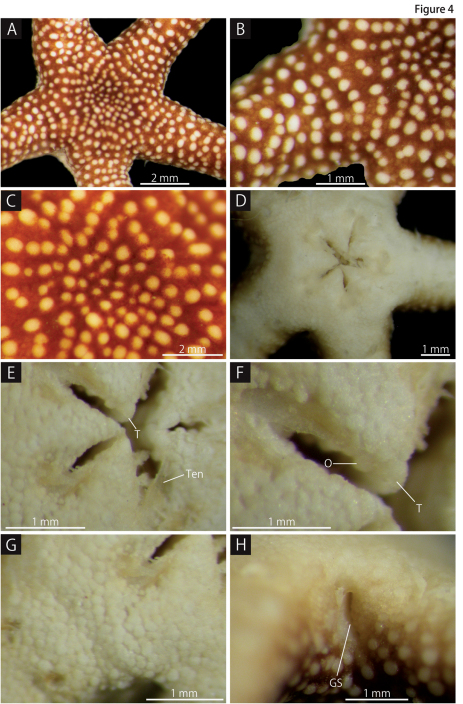
*Squamophis albozosteres* sp. n., holotype (MV F162657). **A** aboral disc and basal portion of the arms **B** periphery of the disc and basal portion of the arm **C** aboral central disc **D** oral disc **E** oral central disc **F** jaws **G** oral periphery of the disc **H** lateral interradius of the disc. Abbreviations: GS - genital slit; O - oral papillae; T - teeth; Ten - Tentacles.

*Disc.* Disc five-lobed with slightly notched interradial edges: lacking evidence of fission (Figs 3, 4A). Aboral surface almost flat, but radial shields and their surrounds slightly tumid, covered by white, slightly domed and round plate-shaped epidermal ossicles and brown, flat and polygonal plate-shaped epidermal ossicles (Fig. 4A–C). Epidermal ossicles covered by a thin skin. White epidermal ossicles forming transverse rows at the aboral disc (Fig. 4A), almost uniform in size on aboral disc, 70–120 µm long, approximately 100 µm thick. Brown epidermal ossicles obscured by skin and cannot observed externally (Fig. 4A–C); relatively large near the periphery, 150–250 µm long, approximately 50 µm thick, and relatively small at the disc center, 100–150 µm long, approximately 50 µm thick. Radial shields completely covered by epidermal ossicles, oblong, approximately 1.2 mm long and 0.6 mm wide, not reaching the center of the disc.

Oral surface of disc entirely covered by only white, flat and polygonal plate-shaped epidermal ossicles, 50–100 µm long and approximately 50 µm thick (Fig. 4D–G). Four to five triangular teeth forming a vertical row on dental plate. Domed granule-shaped oral papillae lying on either side of jaw (Fig. 4F).

Lateral interradial surface of disc nearly vertical, covered by epidermal ossicles similar to those on oral surface (Fig. 4H). Two genital slits (0.6 mm long and 0.3 mm wide) present in each interradius. No distinct ossicles suggesting existence of madreporites or oral plates observed on any oral interradius, and only epidermal ossicles covered these surfaces (Fig. 4H).

**Figure 5. F5:**
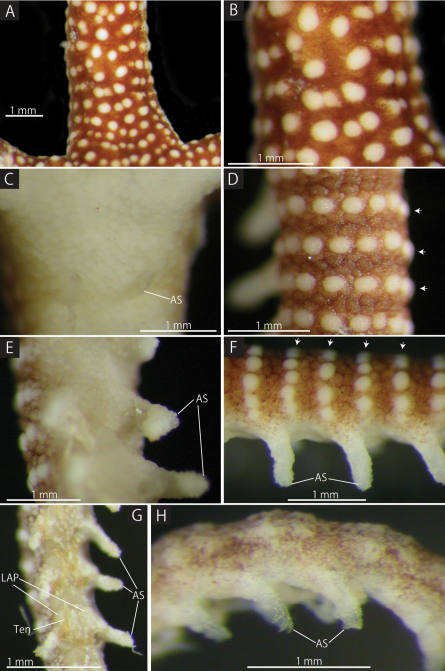
*Squamophis albozosteres* sp. n., holotype (MV F162657). **A, B** aboral basal portion of the arm **C** oral basal portion of the arm **D** aboral middle portion of the arm, each arrow head indicates a row of white ossicles **E** oral middle portion of the arm **F** lateral middle portion of the arm, each arrow head indicates a row of white ossicles **G** oral distal portion of the arm **H** lateral distal portion of the arm. Abbreviations: - AS arm spine; LAP - lateral arm plate; Ten - tentacles.

*Arms.* Arms simple, five in number, no abrupt change in width near the arm base (Fig. 3). The basal portion of the arm 1.4 mm wide and 1.5 mm high, with an arched aboral surface and flattened oral surface. Arms tapering gradually toward the arm tip (Figs 3, 5A, D, G).

The aboral and lateral surface of the base of arms completely covered by white epidermal ossicles, 150–300 µm long, approximately 60 µm thick, and brown epidermal ossicles, 100–300 µm long, approximately 50 µm thick (Fig. 5A, B), similar to those on aboral periphery of disc. Epidermal ossicles on basal portion of arms covered by thin skin. Brown epidermal ossicles obscured by skin, similar to those on aboral surface of disc. Oral surface of the base of arm covered by white epidermal ossicles, 50–100 µm long, approximately 50 µm thick, similar to those on oral surface of disc (Fig. 5C). From basal to middle portion of the arms, the size of plate-shaped epidermal ossicles decreasing on both the aboral and lateral surfaces (Fig. 5D, F), the white domed ones to 100–200 µm and the brown polygonal ones to 100–150 µm. Brown polygonal ones on oral surface decreasing to 50 µm (Fig. 5E). The distal sections of arms covered by scattered granule-shaped epidermal ossicles of 30 µm, finally disappearing near arm tip (Fig. 5G, H).

First to third tentacle pores lacking arm spines; 4th and more distal pores with one arm spine. Arm spines on basal one-third of arm ovoid, minute, approximately one-third to one-half the length of corresponding arm segment (Fig. 5C). Arm spines in middle one-third of arm the same length as corresponding arm segment, bearing fine thorns at their apex (Fig. 5E, F). Arm spines on distal one-third of arm hook-shaped with conspicuous lateral secondary teeth along inner edge (Fig. 5G, H). Length of hook-shaped arm spines gradually decreasing to two-thirds of the corresponding arm segment on distal third of arm, and number of secondary teeth decreasing from two to one. All tentacles pores lacking a sheath around the cylindrical, narrow tube feet (Figs 4E, 5G).

Lateral and ventral arm plates completely concealed by epidermal ossicles over basal to middle portion of arms, but lateral arm plates visible in distal portion of arms (Fig. 5G).

*Color*.Aboral surface of disc brown, with white spots highlighting the domed epidermal ossicles. Pigmentation on aboral distal portion of arms lighter and appearing purple, finally disappearing at the tip (Fig. 5H). Oral side entirely white (Fig. 3).

#### Ossicle morphology of one paratype.

MVF162658: Disc diameter 5.3 mm, arm length at least 200 mm.

**Figure 6. A–K F6:**
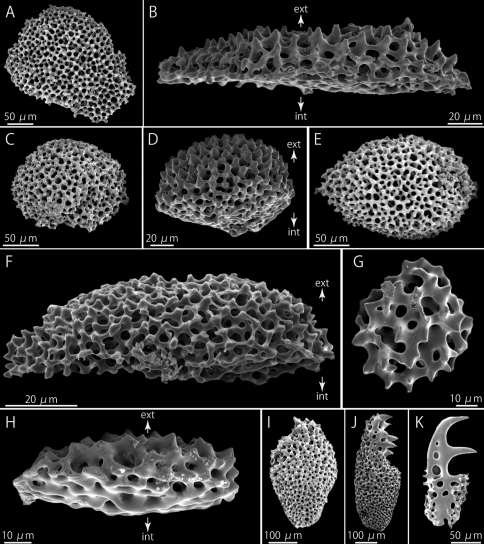
*Squamophis albozosteres* sp. n., paratype(MV F162658). SEM photographs of internal ossicles. **A, B** polygonal plate-shaped epidermal ossicles at the aboral periphery of the disc, external (**A**) and lateral (**B**) views **C, D** domed plate-shaped epidermal ossicles at the periphery of the disc, external (**C**) and lateral (**D**) views **E, F** domed plate-shaped epidermal ossicles on the aboral middle portion of the arm, external (**E**) and lateral (**F**) views **G, H** granule-shaped epidermal ossicles on the oral middle portion of the arm, external (**G**) and lateral (**H**) views **I–K** arm spines from basal (**I**), middle (**J**) and distal (**K**) portion of the arm. Arrows indicate the orientation (**B, D, F, H**): ext - external side; int - internal side.

Flat and polygonal plate-shaped epidermal ossicles at aboral periphery of disc, approximately 236 µm long and 43 µm thick (Fig. 6A, B), the white, round and domed plate-shaped epidermal ossicles approximately 136 µm long and 40 µm thick (Fig. 6C, D). On aboral surface at base of arm, domed ossicles slightly oblong, approximately 226 µm long and 34 µm thick (Fig. 6E, F), whereas the other ossicles flat and round, granule-shaped, 64 µm long and 20 µm thick (Fig. 6G, H).

The radial shields flat and oblong, single-layered, approximately 1.15 mm in length and 0.57 mm in width (Fig. 1B).

Arm spines on basal one-third of arm ovoid (Fig. 6I), in middle cylindrical, bearing fine thorns at tip (Fig. 6J), and distally, they hook shaped with conspicuous secondary teeth along inner edge (Fig. 6K). Number of secondary teeth decreasing gradually to one along distal quarter of arm.

**Figure 7.**
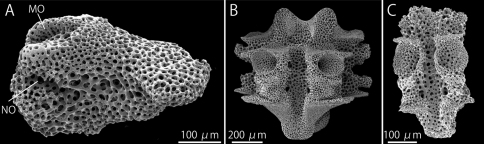
*Squamophis albozosteres* sp. n., paratype(MV F162658). SEM photographs of internal ossicles. **A** lateral arm plate from middle portion of the arm **B, C** vertebrae from middle (**B**) and distal (**C**) portion of the arm, oral views. Abbreviations: MO - muscle opening; NO - nerve opening.

Each lateral arm plate associated with one arm spine and has separate muscle and nerve openings (Martynov 2010) (Fig. 7A). Oral side of each arm vertebra with a longitudinal groove along midline, no oral bridge (Okanishi et al. in press) formed to surround the radial water vessel and nerve (Fig. 7B, C).

#### Variation.

 Although only three specimens have been collected, some morphological variation was observed. The smaller holotype (3.4 mm in disc diameter) has no abrupt reduction in arm thickness, but the basal portion of the arm on the two larger paratypes (5.3 mm and 5.6 mm in disc diameter) are slightly widened. The difference between the three specimens may be due to a difference in their sexual maturity or reproductive state similar to the congener, *Squamophis amamiensis* (Okanishi and Fujita 2009).

#### Distribution.

North-western Australia; 95–108 m. Type locality: off Broome, 100–108 m (Fig. 2).

#### Etymology.

 The specific name is a masculine noun in apposition formed as a compound of Latin words, *albus* (adjective, meaning “whitish”) and a plural form of *zoster* (masculine noun, meaning “ring”), referring to the rings of white plate-shaped dermal ossicles of arms.

#### Remarks.

 *Squamophis albozosteres* sp. n. and its congener, *Squamophis amamiensis*, are similar to each other, however, they can be distinguished by the morphology of the epidermal ossicles on the aboral body and by pigmentation. *Squamophis albozosteres* has conspicuously white, domed and plate-shaped epidermal ossicles on the aboral side of the disc and basal to middle portion of the arms, forming two transverse rows on the lateral and aboral surfaces of each arm segments (Fig. 5F). Whereas *Squamophis amamiensis* has only uniform coloured, flattened and plate-shaped epidermal ossicles on the corresponding surfaces. The aboral body surface of *Squamophis albozosteres* is basically brown with white spots and the tips of the arms are light purple, finally with no color, but that of *Squamophis amamiensis* is uniformly orange or pinkish brown (Okanishi and Fujita 2009).

## Supplementary Material

XML Treatment for Asteroschematidae

XML Treatment for Squamophis

XML Treatment for Squamophis albozosteres

## References

[B1] Byrne M (1994) Ophiuroidea. In: Harrison FW Chia F-S (Eds). Microscopic Anatomy of Invertebrates, Echinodermata: Vol.14. Wiley-Liss, New York: 247-343

[B2] Baker AN (1980) Euryalinid Ophiuroidea (Echinodermata) from Australia, New Zealand, and the south-west Pacific Ocean. New Zealand Journal of Zoology 7: 11-83

[B3] Clark AH (1949) Ophiuroidea of the Hawaiian Islands. Bulletin of the Bernice P. Bishop Museum 195: 3-133

[B4] Döderlein L (1911) Beiträge zur Naturgeschichte Ostasiens. Über japanische und andere Euryalae. Abhandlungen der Bayerischen Akademie der Wissenschaften, II. Suppl.-Bd. 5: 1–123

[B5] Döderlein L (1927) Indopacifische Euryalae. Abhandlungen der Bayerischen Akademie der Wissenschaften 31: 1-105

[B6] Döderlein L (1930) Die Ophiuroiden der deutschen Tiefsee-Expedition. 2. Euryalae. Deutsche Tiefsee-Expedition 1898–1899 22: 347–396

[B7] Farquhar H (1900) On a new species of Ophiuroidea. Transactions of the New Zealand Institute 32: 405

[B8] Fell HB (1960) Synoptic keys to the genera of Ophiuroidea. Zoology publications from Victoria University of Wellington 26: 145-152

[B9] Koehler R (1904) Ophiures de l’Expédition du Siboga. Part I. Ophiures de mer profonde. Siboga-Expedition45a: 1–238

[B10] Lütken CF (1856) Bidrag til Kundskab om Slangestjernerne. II. Oversigt over de vestindiske Ophiurer. Vedenskabelige Meddelelser fra Dansk Naturhistorisk Forening i Kjøbenhavn 7: 1-19

[B11] Lyman T (1869) Preliminary report on the Ophiuridae and Astrophytidae dredged in deep water between Cuba and the Florida Reef, by L. F. de Pourtales, Assist. U. S. Coast Survey. Bulletin of the Museum of Comparative Zoölogy at Harvart College, in Cambridge 1 (10): 309-354

[B12] Lyman T (1883) Reports on the Results of Dredging, under the Supervision of Alexander Agassiz, in the Caribbean Sea in 1878–1879, and along the Atlantic Coast of the United States during the Summer of 1880, by the U. S. Coast Survey Steamer “Blake,” Commander J. R. Bartlett, U. S. N., Commanding. Bulletin of the Museum of Comparative Zoölogy at Harvart College, in Cambridge 10 (6): 227-286

[B13] Martynov A (2010) Reassessment of the classification of the Ophiuroidea (Echinodermata), based on morphological characters. I. General character evaluation and delineation of the families Ophiomyxidae and Ophiacanthidae. Zootaxa 2697: 1-154

[B14] Matsumoto H (1911) Nihon san tedurumoduru rui no ikka ni tsuite [On the Euryalidae in Japan]. Dobutsugaku-zasshi 177: 617-631

[B15] McKnight DG (2000) The marine fauna of New Zealand: Basket-stars and snake-stars (Echinodermata: Ophiuroidea: Euryalinida). National Institute of Water and Atmospheric Research Biodiversity Memoir 115: 1-79

[B16] Mortensen T Stephensen K (1918) Papers from Dr. Mortensen’s Pacific Expedition 1914–1916. II, On a gall-producing parasitic copepod, infesting an ophiuroid. Vidensk Medd fra dansk Naturh Foren Kjøbenhavn 69: 263-275

[B17] Mortensen T (1925) On some echinoderms from South Africa. *The Annals and* Magazine of Natural History Including Zoology, Botany, and Geology, Ninth Series 16: 146-155

[B18] Mortensen T (1933) Studies of Indo-Pacific euryalids. Videnskabelige Meddelelser fra Dansk Naturhistorisk Forening i Kjøbenhavn 96: 1-75

[B19] Müller J Troschel FH (1842) System der Asteriden. Braunschweig, Papier, Druck und Verlag von Friedrich Vieweg und Sohn, 134 pp.

[B20] Okanishi M Fujita T (2009) A new species of *Asteroschema* (Echinodermata: Ophiuroidea: Asteroschematidae) from southwestern Japan. Species Diversity 14: 115-129

[B21] Okanishi M Fujita T (2011a) A taxonomic review of the genus *Astrocharis* Koehler (Echinodermata: Ophiuroidea: Asteroschematidae), with a description of a new species. Zoological Science 28 (2): 148-157 doi: 10.2108/zsj.28.148 21303207 10.2108/zsj.28.148

[B22] Okanishi M Fujita T (2011b) Ophiuroids of the order Euryalida (Echinodermata) from Hachijojima Island and Ogasawara Islands, Japan. Memoires of the National Museum of Nature and Science 47: 368-385

[B23] Okanishi M O’Hara TD Fujita T (in press) Molecular phylogeny of the order Euryalida (Echinodermata: Ophiuroidea), based on mitochondrial and nuclear ribosomal genes. Molecular Phylogenetics and Evolution. 10.1016/j.ympev.2011.07.003 21798356

[B24] Pallas PS (1788) Maria varia nova et rariora. Nova Acta Academiae Scientarum Imperialis Petropolitanae 2: 229-240

[B25] Studer T (1884) Verzeichniss der während der Reise S.M.S. Gazelle um die Erde 1874–1876 gesammelten Asteriden und Euryaliden. Abhandlungen der Preussischen Akademie der Wissenschaften 2: 1-64

[B26] Verrill AE (1899) Report on the Ophiuroidea collected by the Bahama Expedition in 1893. Bulletin from the Laboratory of Natural History of the State University of Iowa 5 (1): 1-86

